# Co-occurring divergent arbitrium receptors outside canonical prophage regions in bacterial genomes

**DOI:** 10.1007/s10142-026-02109-2

**Published:** 2026-11-09

**Authors:** José L. Balcázar

**Affiliations:** Catalan Institute for Water Research (ICRA-CERCA), 17003 Girona, Spain

**Keywords:** Arbitrium signaling, AimR-like receptors, Sequence divergence, Prophage regions, Genomic organization

## Abstract

Arbitrium signaling enables peptide-mediated communication among bacteriophages and regulates lysis-lysogeny decisions through diverse genetic architectures. Here, we identified two divergent AimR-like receptors in each of the alkaliphilic bacteria *Alkalihalobacterium alkalinitrilicum* DSM 22532 and *Caldalkalibacillus mannanilyticus* JCM 10596, with within-genome amino acid identities of 30.6% and 27.4%, respectively. All four receptors occurred outside predicted prophage regions, with distinct genomic neighborhoods and a putative *aimP*-like gene adjacent to one receptor. Despite substantial sequence divergence, structural comparisons revealed conservation within aligned regions of the AimR fold. Associations with mobile genetic elements suggest possible genomic mobility, although the evolutionary origins and functional roles of these receptors remain unresolved. These findings highlight the diversity of co-occurring AimR-like receptors in bacterial genomes.

**Supplementary Information:**

The online version contains supplementary material available at https://doi.org/10.1007/s10142-026-02109-2.

Bacteriophages use arbitrium signaling systems to coordinate lysis-lysogeny decisions through peptide-mediated communication (Erez et al. 2017). Initially characterized as a three-component system involving the signaling peptide AimP, the receptor AimR, and the regulatory element AimX, arbitrium signaling is now recognized to encompass more diverse genetic architectures and regulatory mechanisms (Erez et al. 2017; Brady et al. 2023; Kabel et al. 2026). Comparative genomic studies have also identified AimR homologs outside recognizable mobile genetic elements and without associated AimP peptides (Stokar-Avihail et al. 2019; Felipe-Ruiz et al. 2022). Recent experimental evidence has further demonstrated that arbitrium signaling can occur between distinct phage lineages through non-cognate peptide-receptor interactions (Gallego-Del-Sol et al. 2026; Manley et al. 2026; Lomeli-Ortega and Balcázar 2026). However, the genomic organization and structural conservation of co-occurring divergent AimR-like receptors outside recognizable prophage regions remain insufficiently characterized.

To investigate the genomic distribution of arbitrium-related components, protein similarity searches were performed against the NCBI non-redundant protein sequence database (nr), using the AimR (CAB14004.1) and AimP (CAB14003.1) proteins from *Bacillus subtilis* subsp. *subtilis* str. 168 (GenBank accession AL009126.3) as reference sequences. Initial searches were conducted using BLASTP, followed by two iterations of PSI-BLAST for both proteins to identify more divergent homologs, with a maximum of 500 sequences selected for each subsequent iteration. Searches for *aimX* were initially performed using BLASTN, followed by manual inspection of the genomic regions surrounding candidate *aimR*-like genes. Following the identification of two AimR-like homologs within the same bacterial genome, subsequent analyses focused on their sequence divergence, genomic organization, and association with prophages and other mobile genetic elements. Candidate sequences and their surrounding genomic regions were manually inspected. Prophage regions were predicted using PHASTEST (Wishart et al. 2023), whereas putative horizontally transferred regions were identified using Alien Hunter (Vernikos and Parkhill 2006).

Comparative genomic analysis of *B. subtilis* subsp. subtilis str. 168 and *B. subtilis* KRS015 (NZ_CP125762.1) revealed differences in the genomic organization of arbitrium-associated loci (Fig. 1A). In strain 168, the arbitrium locus was located within a predicted prophage region. In contrast, KRS015 contained two *aimR-aimP-aimX* modules within a 269.2-kb putative horizontally transferred region identified using Alien Hunter. Although two prophage regions were predicted in KRS015 using PHASTEST, neither encompassed these arbitrium modules. The two AimR proteins encoded by these modules shared 100% amino acid sequence identity, providing a reference for comparison with the divergent AimR-like homologs identified in alkaliphilic bacteria.

**Fig. 1:**
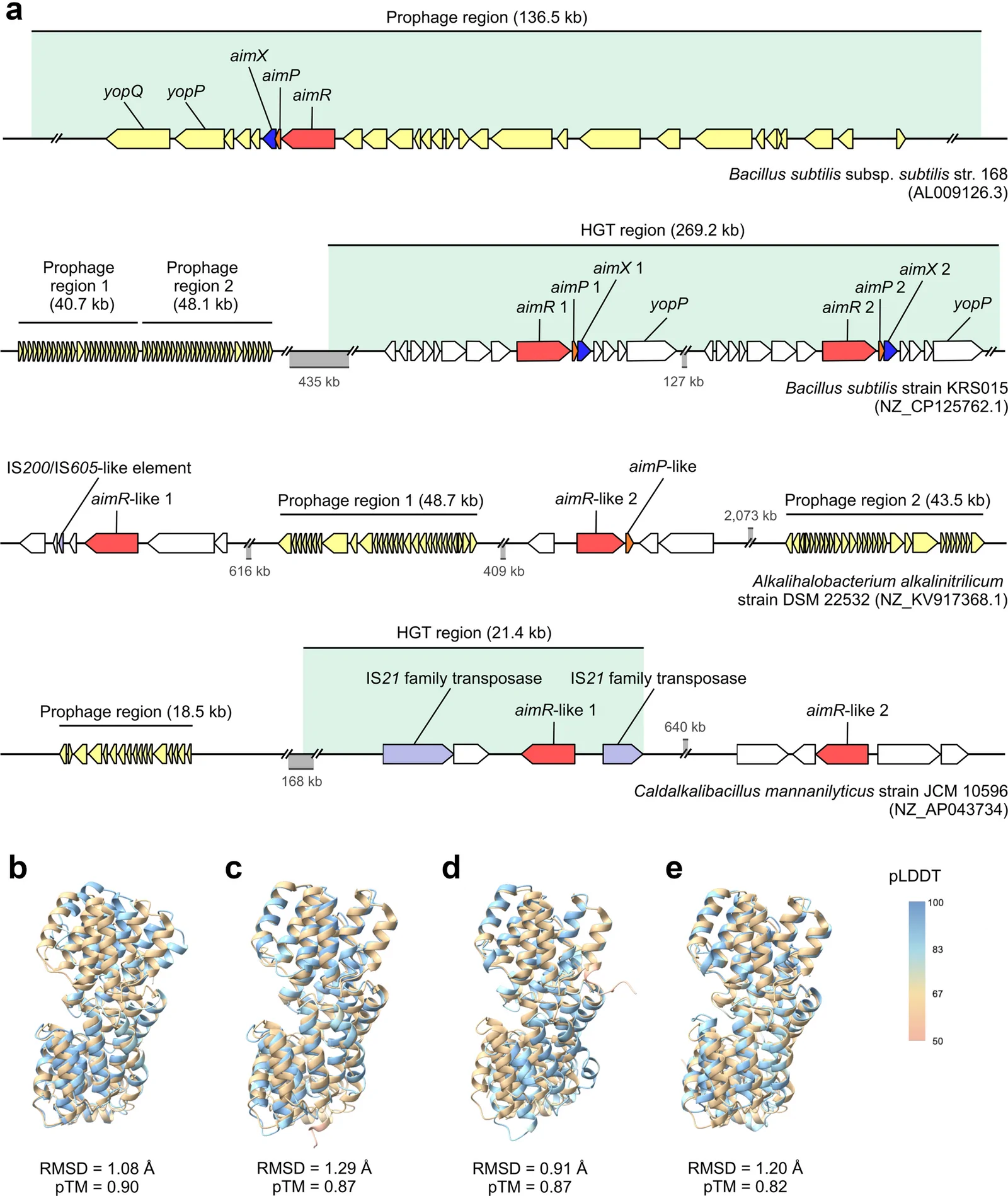
Genomic organization and structural comparison of canonical AimR and divergent AimR-like receptors. **a** Genomic organization of arbitrium-associated loci in *Bacillus subtilis* subsp. *subtilis* str. 168, *B. subtilis* KRS015, *Alkalihalobacterium alkalinitrilicum* DSM 22532, and *Caldalkalibacillus mannanilyticus* JCM 10596. In *B. subtilis* 168, the *aimR-aimP-aimX* module is located within a prophage region, whereas KRS015 contains two such modules within a 269.2-kb putative horizontally transferred region. The two AimR proteins in KRS015 share 100% amino acid sequence identity. Both alkaliphilic species harbor two divergent *aimR*-like homologs outside predicted prophage regions. In *A. alkalinitrilicum*, one homolog is located near an IS*200*/IS*605*-like element, whereas the other is adjacent to a putative *aimP*-like gene. In *C. mannanilyticus*, one homolog is located within a 21.4-kb putative horizontally transferred region containing IS*21* family transposases. Prophage regions were predicted using PHASTEST (Wishart et al. 2023), and putative horizontally transferred regions were identified using Alien Hunter (Vernikos and Parkhill 2006). Yellow arrows indicate prophage-associated genes; red arrows, *aimR* or *aimR*-like genes; orange arrows, *aimP* or putative *aimP*-like genes; blue arrows, *aimX*; purple arrows, transposases; and white arrows, neighboring genes. Double slashes indicate omitted genomic intervals, with the corresponding distances indicated in kilobases (kb). Genomic features are shown schematically and are not drawn to scale. **b**–**e** Structural superpositions of the experimentally determined SPβ AimR receptor (PDB: 5ZW5, chain A) with AlphaFold 3-predicted AimR-like proteins from *A. alkalinitrilicum* AimR-like 1b, *A. alkalinitrilicum* AimR-like 2 **c**, *C. mannanilyticus* AimR-like 1 **d**, and *C. mannanilyticus* AimR-like 2 **e**. Structures were aligned using the MatchMaker algorithm implemented in UCSF ChimeraX (Pettersen et al. 2021). The experimentally determined SPβ AimR structure is shown in beige, whereas the predicted AimR-like structures are colored according to their per-residue predicted local distance difference test (pLDDT) scores, using a shared color scale (50–100). Root-mean-square deviation (RMSD) values, calculated after iterative pruning of structurally divergent atom pairs, and predicted template modeling (pTM) scores are indicated for each superposition. The structural comparisons reveal conservation within aligned regions despite substantial sequence divergence (Supplementary Table S1)

Further analysis identified two AimR-like homologs in each of the alkaliphilic bacteria *Alkalihalobacterium alkalinitrilicum* DSM 22532 (NZ_KV917368.1) and *Caldalkalibacillus mannanilyticus* JCM 10596 (NZ_AP043734) (Fig. 1A). All four homologs were located outside the prophage regions predicted by PHASTEST, although their genomic neighborhoods differed. In *A. alkalinitrilicum*, *aimR*-like 1 was located near an IS*200*/IS*605*-like element, whereas *aimR*-like 2 was immediately adjacent to a putative *aimP*-like gene. The corresponding protein was analyzed using SignalP 6.0 (Teufel et al. 2022), which predicted a Sec/SPI signal peptide with a cleavage site between residues 24 and 25. No recognizable *aimX* gene was identified in the surrounding genomic regions. In *C. mannanilyticus*, *aimR*-like 1 was located within a 21.4-kb putative horizontally transferred region predicted by Alien Hunter, near genes encoding IS*21* family transposases, whereas *aimR*-like 2 was located outside this region. Neither receptor was associated with a recognizable *aimP*-like or *aimX* gene. These observations reveal distinct genomic arrangements among the co-occurring AimR-like homologs, including the presence of a putative *aimP*-like gene adjacent to one receptor, but no recognizable *aimX* genes in their genomic neighborhoods.

Sequence comparisons revealed substantial divergence between the co-occurring AimR-like homologs, with amino acid sequence identities of 30.6% in *A. alkalinitrilicum* and 27.4% in *C. mannanilyticus*. Phylogenetic analysis was performed using MUSCLE for sequence alignment (Edgar 2004) and the neighbor-joining method implemented in MEGA12 (Kumar et al. 2024), with 1,000 bootstrap replicates. The resulting phylogeny illustrated the sequence diversity of the identified AimR-like homologs relative to the reference AimR proteins (Supplementary Figure S1).

To further assess the structural similarity between the identified AimR-like proteins and canonical AimR receptors, three-dimensional structures were predicted using AlphaFold 3 (Abramson et al. 2024) and aligned against the experimentally determined structure of the SPβ AimR receptor (PDB: 5ZW5) (Wang et al. 2018) using the MatchMaker algorithm implemented in UCSF ChimeraX (Pettersen et al. 2021). The predicted structures exhibited high confidence, with mean pLDDT values ranging from 88.84 to 92.21. Despite substantial amino acid sequence divergence, all four AimR-like proteins exhibited an α-helical architecture resembling that of the reference AimR receptor (Fig. 1B–E). Structural superposition yielded RMSD values ranging from 0.91 to 1.29 Å after pruning structurally discordant atom pairs, whereas RMSD values calculated across all initially aligned atom pairs ranged from 3.25 to 4.87 Å (Supplementary Table S1). These results indicate structural similarity within the retained aligned regions, with larger deviations observed when all aligned atom pairs were considered. Protein domain analyses using InterPro (Blum et al. 2025), Pfam (Mistry et al. 2021), CDD (Wang et al. 2023), and HHpred (Zimmermann et al. 2018) further supported their annotation as AimR-like proteins.

The heterogeneous genomic contexts of the identified AimR-like homologs suggest possible associations with mobile genetic elements and horizontal gene transfer. The proximity of some *aimR*-like genes to insertion sequence elements, together with the localization of one homolog within a putative horizontally transferred region, is consistent with the possibility that genomic mobility has contributed to their distribution. However, these associations were not observed for all four receptors, and the available genomic evidence does not establish whether the aimR-like genes underwent horizontal transfer. Furthermore, their location outside prophage regions predicted by PHASTEST does not exclude an association with degraded prophages or other unrecognized mobile genetic elements.

Previous studies have identified AimR homologs outside recognizable mobile genetic elements, including receptors lacking adjacent AimP-encoding genes (Stokar-Avihail et al. 2019; Felipe-Ruiz et al. 2022). The present findings extend these observations by documenting the coexistence of markedly divergent AimR-like homologs within individual bacterial genomes, despite their structural resemblance to canonical AimR receptors. Although a putative *aimP*-like gene was identified adjacent to one receptor, its predicted signal peptide does not establish a role in arbitrium signaling, and no recognizable *aimX* genes were detected in the genomic neighborhoods analyzed. The possibility that these receptors recognize peptides encoded elsewhere or produced by other microorganisms is consistent with recent evidence of non-cognate peptide-receptor interactions among arbitrium systems (Gallego-Del-Sol et al. 2026; 2026). Nevertheless, the signaling capacity and regulatory functions of the identified receptors remain unknown.

Together, these findings highlight the diversity of genomic arrangements and the structural similarities of co-occurring, sequence-divergent AimR-like receptors outside prophage regions predicted by PHASTEST. Their evolutionary origins and functional significance remain unresolved. Future studies integrating comparative genomics, peptide-binding assays, and transcriptional analyses will be necessary to determine whether these receptors recognize distinct or shared signaling peptides or perform alternative regulatory functions.

## Supplementary Information

Below is the link to the electronic supplementary material.

Supplementary file1 Phylogenetic relationships among AimR and AimR-like proteins. Neighbor-joining phylogenetic tree constructed from amino acid sequences aligned using MUSCLE [12], with 1,000 bootstrap replicates in MEGA12 [13]. The four AimR-like homologs identified in Alkalihalobacterium alkalinitrilicum DSM 22532 and Caldalkalibacillus mannanilyticus JCM 10596 are highlighted in bold, whereas the reference AimR protein from Bacillus subtilis subsp. subtilis str. 168 (CAB14004.1) is highlighted in grey. Bootstrap support values greater than 60% are indicated at the nodes. The scale bar represents amino acid substitutions per site. (PDF 62 KB)

Supplementary file2 Structural comparison of canonical AimR and AimR-like proteins. Structural alignments were performed against the experimentally determined SPβ AimR receptor (PDB: 5ZW5, chain A) using the MatchMaker algorithm implemented in UCSF ChimeraX [16] (DOCX 21 KB)

## Data Availability

No datasets were generated or analysed during the current study.
