# Supplementary material for: Co-occurring divergent arbitrium receptors outside canonical prophage regions in bacterial genomes
Source: Funct Integr Genomics. 2026 Nov 9;26(1):330. doi: 10.1007/s10142-026-02109-2 (PMC13748020; doi:10.1007/s10142-026-02109-2)
Supplement: Supplementary file 1 [file 10142_2026_2109_MOESM1_ESM.pdf]

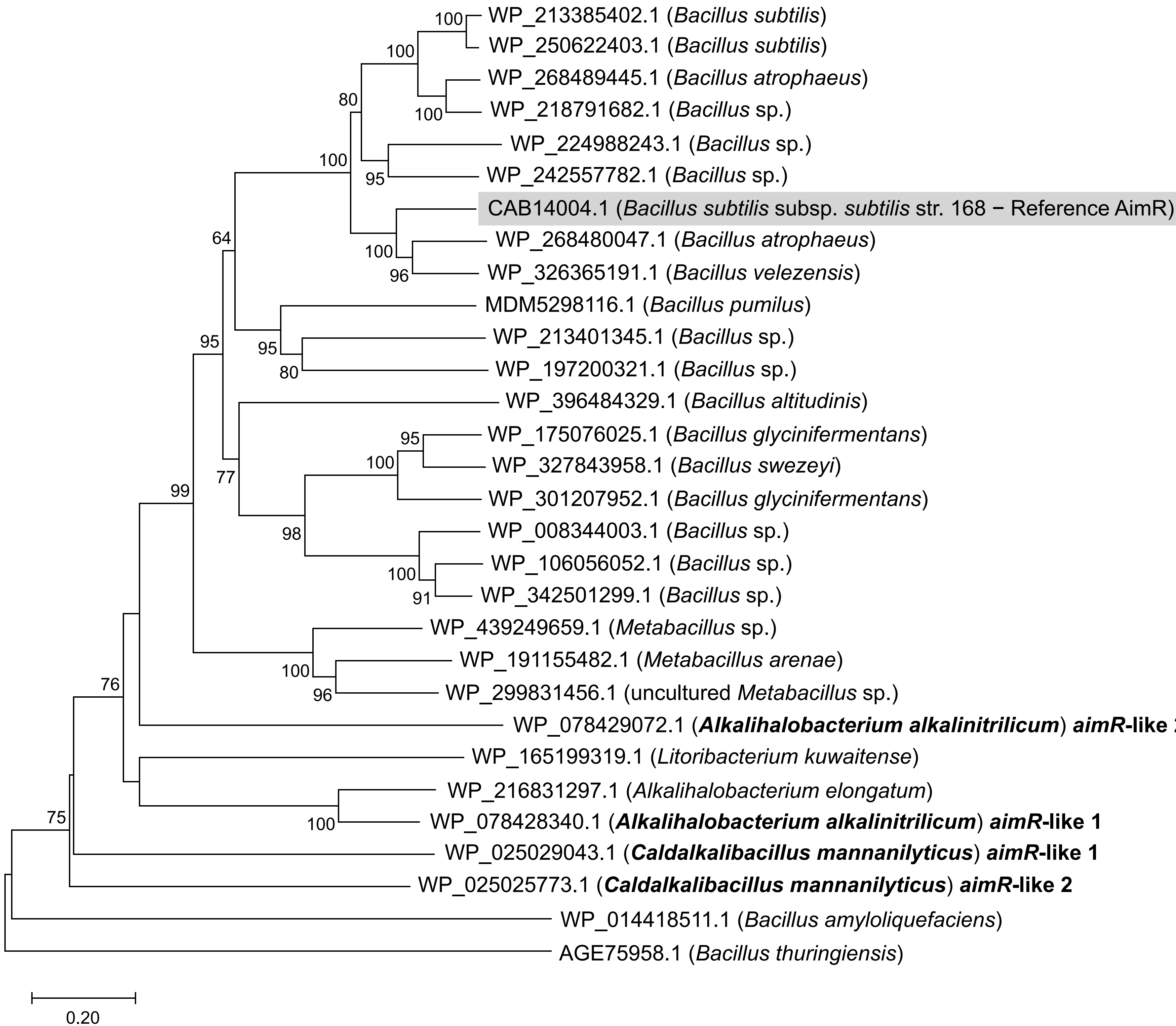
WP_213385402.1 (Bacillus subtilis)
WP_250622403.1 (Bacillus subtilis)
WP_268489445.1 (Bacillus atrophaeus)
WP_218791682.1 (Bacillus sp.)
WP_224988243.1 (Bacillus sp.)
WP_242557782.1 (Bacillus sp.)
CAB14004.1 (Bacillus subtilis subsp. subtilis str. 168 − Reference AimR)
WP_268480047.1 (Bacillus atrophaeus)
WP_326365191.1 (Bacillus velezensis)
MDM5298116.1 (Bacillus pumilus)
WP_213401345.1 (Bacillus sp.)
WP_197200321.1 (Bacillus sp.)
WP_396484329.1 (Bacillus altitudinis)
WP_175076025.1 (Bacillus glycinifermentans)
WP_327843958.1 (Bacillus swezeyi)
WP_301207952.1 (Bacillus glycinifermentans)
WP_008344003.1 (Bacillus sp.)
WP_106056052.1 (Bacillus sp.)
WP_342501299.1 (Bacillus sp.)
WP_439249659.1 (Metabacillus sp.)
WP_191155482.1 (Metabacillus arenae)
WP_299831456.1 (uncultured Metabacillus sp.)
WP_078429072.1 (Alkalihalobacterium alkalinitrilicum) aimR-like 2
WP_165199319.1 (Litoribacterium kuwaitense)
WP_216831297.1 (Alkalihalobacterium elongatum)
WP_078428340.1 (Alkalihalobacterium alkalinitrilicum) aimR-like 1
WP_025029043.1 (Caldalkalibacillus mannanilyticus) aimR-like 1
WP_025025773.1 (Caldalkalibacillus mannanilyticus) aimR-like 2
WP_014418511.1 (Bacillus amyloliquefaciens)
AGE75958.1 (Bacillus thuringiensis)
0.20
