# Supplementary material for: Co-occurring divergent arbitrium receptors outside canonical prophage regions in bacterial genomes
Source: Funct Integr Genomics. 2026 Nov 9;26(1):330. doi: 10.1007/s10142-026-02109-2 (PMC13748020; doi:10.1007/s10142-026-02109-2)
Supplement: Supplementary file 2 [file 10142_2026_2109_MOESM2_ESM.docx]

**Supplementary Table S1. Structural comparison of canonical AimR and AimR-like proteins.** Structural alignments were performed against the experimentally determined SPβ AimR receptor (PDB: 5ZW5, chain A) using the MatchMaker algorithm implemented in UCSF ChimeraX [16].

| **Receptor** | **Source organism** | **Atom pairs after pruning** | **Total aligned atom pairs** | **RMSD after pruning (Å)** | **RMSD across all aligned pairs (Å)** |
| --- | --- | --- | --- | --- | --- |
| AimR-like 1 | *A. alkalinitrilicum* DSM 22532 | 210 | 380 | 1.08 | 4.07 |
| AimR-like 2 | *A. alkalinitrilicum* DSM 22532 | 226 | 337 | 1.29 | 3.35 |
| AimR-like 1 | *C. mannanilyticus* JCM 10596 | 108 | 379 | 0.91 | 4.87 |
| AimR-like 2 | *C. mannanilyticus* JCM 10596 | 207 | 372 | 1.20 | 3.25 |

RMSD values were calculated using Cα atom pairs. RMSD after pruning was calculated after excluding structurally discordant atom pairs using the default MatchMaker iteration cutoff of 2 Å. RMSD across all aligned pairs was calculated before pruning. Differences in the number of retained atom pairs reflect variation in the extent of structural superposition.
